# Anatomical–Functional Dissociation in Diabetic Macular Edema: Five-Year Outcomes of Treat-and-Extend Versus Pro Re Nata Anti-VEGF Regimens

**DOI:** 10.3390/jcm15166450

**Published:** 2026-08-20

**Authors:** Burhan Başkan, Yusuf Evcimen

**Affiliations:** Department of Ophthalmology, Van Training and Research Hospital, University of Health Sciences, 65300 Van, Turkey; yusufevcimen23@gmail.com

**Keywords:** diabetic macular edema, anti-vascular endothelial growth factor, treat-and-extend, pro re nata, intravitreal injections

## Abstract

**Objectives**: To determine whether the superior anatomical control achieved with a treat-and-extend (T&E) anti-VEGF regimen translates into better five-year visual outcomes than a pro re nata (PRN) regimen in treatment-naïve center-involving diabetic macular edema (CI-DME), and to characterize the anatomical–functional relationship. **Methods**: In this retrospective propensity-score–matched survivor cohort, one eye per patient was analyzed. One-to-one nearest-neighbor matching balanced 14 baseline covariates. Longitudinal best-corrected visual acuity (BCVA) and central subfield thickness (CST) were analyzed using linear mixed-effects models with matched-pair clustering. Absence of a clinically meaningful visual difference was evaluated using two one-sided tests (TOST) with a prespecified ±5-letter equivalence margin. The anatomical–functional relationship was assessed by segmented regression. Sensitivity analyses included inverse probability of treatment weighting, doubly robust estimation, inverse probability of censoring weighting, best-/worst-case imputation, interval-censored recurrence modeling, and E-value analysis. **Results**: Both regimens improved BCVA, with no clinically meaningful difference at five years (T&E +6.2 ± 14.6 vs. PRN +6.9 ± 14.0 letters; difference −0.7 letters; 90% CI, −2.4 to 1.0; TOST *p* < 0.001). T&E achieved greater CST reduction (−172.3 vs. −114.2 µm; difference −58.1 µm; *p* < 0.001), higher dry-macula rates (73.8% vs. 59.5%; *p* < 0.001), fewer recurrences (2.2 vs. 3.9; *p* < 0.001), and fewer monitoring-only visits (14.6 vs. 28.4; *p* < 0.001), but required 53% more injections (25.1 vs. 16.4; *p* < 0.001). Segmented regression identified a breakpoint at 148 µm CST reduction; additional thinning beyond this threshold was not associated with further visual improvement. Baseline BCVA, ellipsoid zone disruption, and diabetic retinopathy severity, but not treatment regimen, independently predicted five-year vision. Results were consistent across sensitivity analyses. **Conclusions**: Among patients completing five years of therapy, additional anatomical drying beyond an exploratory, cohort-specific breakpoint of approximately 150 µm CST reduction was not associated with further measurable visual gain in this observational cohort. Regimen selection should therefore reflect treatment burden, monitoring requirements, and patient preference rather than anticipated visual superiority. Because the cohort included only patients completing five years of follow-up, these findings apply to adherent patients and should not be generalized to unselected populations.

## 1. Introduction

Diabetic macular edema (DME) remains a leading cause of vision impairment among working-age adults, and its burden is expanding with the global diabetes epidemic [1,2]. Chronic hyperglycemia drives overexpression of vascular endothelial growth factor (VEGF), disruption of the blood–retinal barrier, and accumulation of intraretinal and subretinal fluid, with progressive and partly irreversible injury to the neurosensory retina [3,4].

Intravitreal anti-VEGF therapy transformed this landscape. The VIVID/VISTA program and DRCR Retina Network Protocol T established the efficacy of aflibercept, bevacizumab, and ranibizumab in center-involving DME (CI-DME) [5,6], and dual angiopoietin-2/VEGF-A inhibition has since extended achievable dosing intervals further [7]. What has not been settled is how a fixed initial gain should be maintained over years. Two flexible strategies dominate: pro re nata (PRN), which is reactive and requires frequent monitoring, and treat-and-extend (T&E), which is proactive and trades injections for fewer monitoring-only visits [8].

Randomized comparisons exist but are short. TREX-DME showed that T&E ranibizumab performed comparably to monthly dosing at two years, an aflibercept T&E regimen was non-inferior to monthly dosing at two years, and a meta-analysis of 2346 eyes found similar 12- and 24-month visual outcomes for T&E and PRN with fewer visits but more injections under T&E. Beyond two years the evidence is almost entirely observational [9,10,11,12]. The Protocol T extension reported a mean gain of 7.4 letters at five years despite substantial loss of the two-year advantage, Wecker et al. reported approximately +5 letters at five years under PRN, and registry data show smaller gains still [13,14].

A more fundamental question is rarely addressed. Anti-VEGF regimens are compared, and treatment targets are set, on the implicit assumption that more complete fluid resolution yields better vision. If the relationship between anatomical drying and visual gain saturates, then a regimen that dries the macula more completely may deliver no functional dividend while consuming substantially more drug, chair time, and money. Post hoc analyses in neovascular age-related macular degeneration have hinted at such a ceiling, and the fluid–function relationship in DME is known to be imperfect, but the shape of that relationship has not been formally estimated over a five-year horizon in a regimen comparison [15].

We therefore compared five-year visual and anatomical outcomes between T&E and PRN in a propensity-score–matched cohort of treatment-naïve CI-DME patients who completed 60 months of continuous follow-up, and we modeled the anatomical–functional relationship directly.

We state at the outset that conditioning on five-year survival is a deliberate design choice with a specific cost: the cohort is adherent by construction, and all estimates are conditional on that selection. The 383 excluded patients (29.9% of the screened population) were unable to complete five years of follow-up for various reasons (treatment discontinuation, loss to follow-up, migration, death, or incomplete records). Our findings do not apply to patients who discontinue treatment or are lost to follow-up, and should not be interpreted as evidence against the use of T&E in non-adherent or high-risk populations.

## 2. Methods

### 2.1. Study Design, Setting, and Ethics

This was a retrospective, propensity-score–matched survivor cohort study conducted at the Department of Ophthalmology, SBÜ Van Training and Research Hospital, a tertiary referral center serving Eastern Anatolia, Türkiye. Patients initiated therapy between May 2016 and May 2021; because 60 months of continuous follow-up were required, clinical observation extended to May 2026. Data were extracted retrospectively from electronic and paper records in June 2026, after ethics approval.

The study was approved by the Van Training and Research Hospital Non-Interventional Clinical Research Ethics Committee (approval no. GOKAEK/2026-06-03) and adhered to the Declaration of Helsinki and to Turkish Personal Data Protection Law no. 6698. Given the fully retrospective design and the use of a de-identified dataset, the requirement for written informed consent was waived by the committee.

### 2.2. Participants

Consecutive treatment-naïve patients with CI-DME who initiated either a T&E or a PRN anti-VEGF regimen during the enrollment window were identified (*n* = 1283). Inclusion criteria were: (1) CI-DME with central subfield thickness (CST) ≥ 300 µm on spectral-domain optical coherence tomography (SD-OCT), measured from the internal limiting membrane to Bruch membrane (Spectralis convention); (2) type 1 or type 2 diabetes mellitus; (3) baseline BCVA between 20 and 85 ETDRS letters; (4) complete baseline records; and (5) completion of 60 months of follow-up.

Exclusion criteria were: (1) visually significant media opacity at baseline; (2) pars plana vitrectomy within the preceding six months; (3) concurrent systemic anti-VEGF therapy; (4) active ocular surface disease; (5) more than two consecutive missed scheduled visits; (6) prior anti-VEGF therapy for any retinal disease; (7) baseline macular ischemia on fluorescein angiography, epiretinal membrane, or vitreomacular traction; and (8) cataract surgery within the first six months, or lens opacity precluding reliable BCVA measurement at more than two consecutive visits.

**Figure 1:**
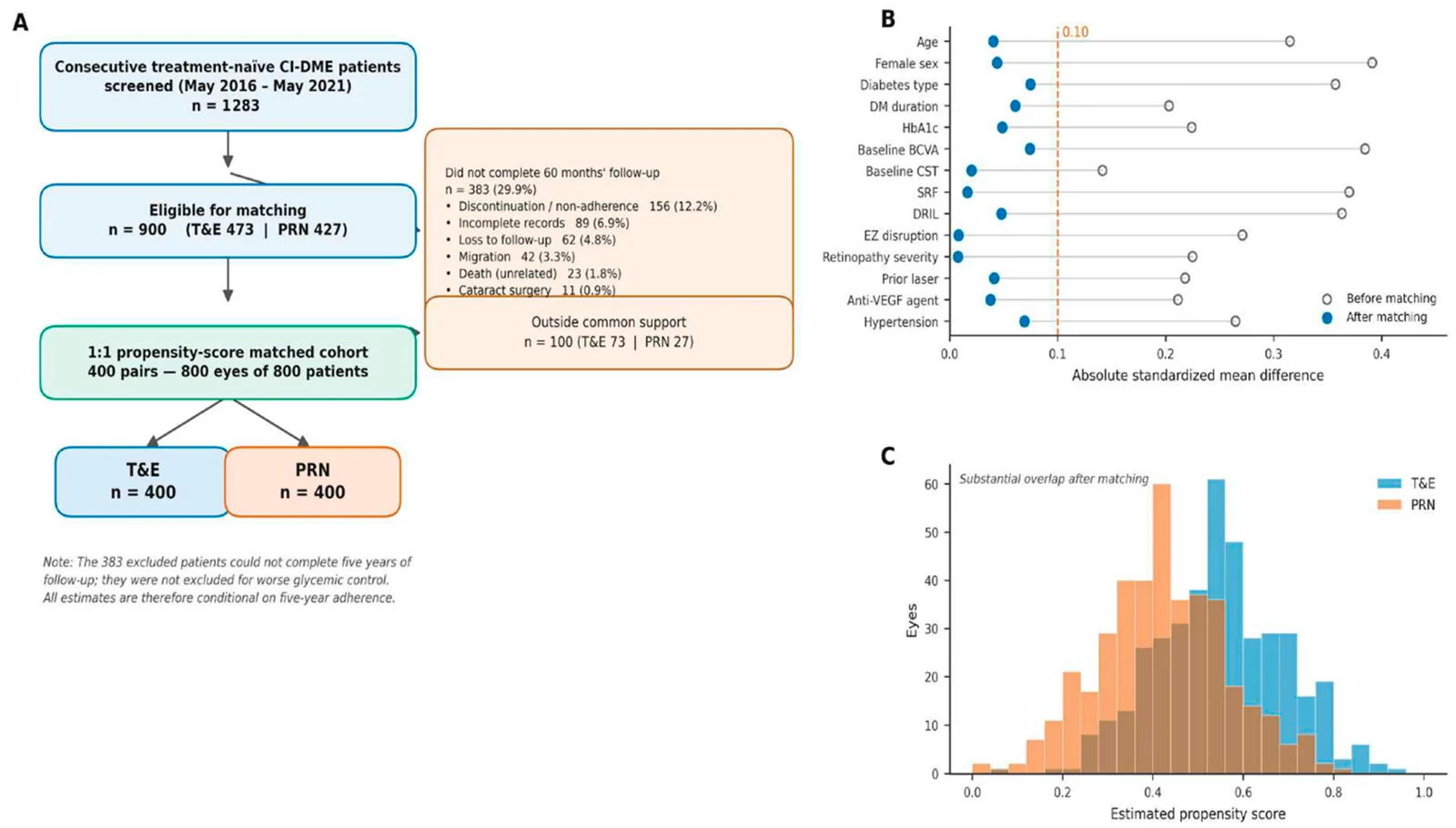
Participant flow and propensity-score matching. (**A**) Flow diagram. Of 1283 screened patients, 383 (29.9%) did not complete 60 months of follow-up and 100 fell outside the region of common support, leaving 400 matched pairs (800 eyes). (**B**) Love plot of absolute standardized mean differences for the 14 baseline covariates before and after matching; all values fell below the 0.10 threshold after matching. (**C**) Overlapping propensity score distributions after matching. The 383 excluded patients could not complete five years of follow-up; they were not excluded because of worse glycemic control.

A total of 383 patients (29.9%) failed the 60-month completion requirement: treatment discontinuation or documented non-adherence (*n* = 156, 12.2%), incomplete records (*n* = 89, 6.9%), loss to follow-up (*n* = 62, 4.8%), migration out of the catchment area (*n* = 42, 3.3%), death from causes unrelated to therapy (*n* = 23, 1.8%), and cataract surgery precluding accurate visual assessment (*n* = 11, 0.9%). Nine hundred patients remained eligible for matching (Figure 1).

These 383 excluded patients were unable to complete five years of follow-up for these reasons. They were not excluded because of worse glycemic control; rather, their inability to maintain treatment and follow-up is the reason for exclusion. This distinction is critical for interpreting the study findings.

### 2.3. Treatment Protocols

Regimen selection followed a documented institutional algorithm incorporating baseline disease severity, anticipated adherence, travel distance, patient preference, and physician judgement. This allocation is non-random and is the principal threat to internal validity; it is addressed by propensity-score matching, by four alternative causal estimators, and by quantitative bias analysis (below).

T&E. Three consecutive monthly loading injections were given. Thereafter, if the macula was dry on SD-OCT and BCVA was stable or improved, the interval was extended in two-week increments to a maximum of 12 weeks. On recurrence (CST increase ≥ 50 µm above the individual post-loading nadir, or new intraretinal or subretinal fluid), the interval was shortened by two weeks. Every scheduled T&E visit was a treatment visit; monitoring-only visits occurred when treatment was withheld for an intercurrent reason, at the protocol-mandated annual comprehensive assessment, or at unscheduled symptomatic presentations.

PRN. Patients were scheduled for monthly evaluation throughout follow-up. Treatment was administered when disease activity was detected (BCVA decrease ≥ 5 letters attributable to DME, CST increase ≥ 50 µm, or new intraretinal or subretinal fluid); otherwise the patient returned the following month. Attendance was incomplete: PRN patients attended a mean of 44.8 ± 9.1 of 60 scheduled visits (74.7%), against 39.7 ± 7.4 total attended visits in the T&E arm.

Two vitreoretinal specialists made all treatment decisions. Because only two clinicians contributed, physician was modeled as a single fixed-effect indicator rather than a random effect; a variance component is not estimable from two clusters, and no intraclass correlation is reported.

Patients received ranibizumab 0.5 mg/0.05 mL (Lucentis; Novartis Pharma AG, Basel, Switzerland), aflibercept 2.0 mg/0.05 mL (Eylea; Bayer AG, Leverkusen, Germany), or bevacizumab 1.25 mg/0.05 mL (Avastin; F. Hoffmann-La Roche Ltd., Basel, Switzerland) according to clinical indication, national reimbursement rules, and patient preference. Bevacizumab was used off-label with documented informed consent. Agent was a matching covariate. Switching occurred in 54 patients (6.8%) and did not differ between arms (T&E 7.0% vs. PRN 6.5%; *p* = 0.78); switching was modeled as a time-varying covariate and a switcher-excluded analysis is reported.

### 2.4. Outcome Measures

The primary outcome was mean change in BCVA from baseline to year 5, measured on ETDRS charts at 4 m (1 m for low vision) by certified technicians under standardized luminance (85 cd/m^2^) with best refractive correction.

Secondary outcomes were: change in CST; proportion with complete resolution of intraretinal and subretinal fluid (“dry macula”); number of recurrences; time to first recurrence; total injections; total monitoring-only visits; proportion gaining or losing ≥ 15 letters; adjunctive and rescue therapy (focal/grid laser, panretinal photocoagulation, intravitreal dexamethasone implant, vitrectomy); cataract surgery; and ocular and systemic adverse events.

SD-OCT was performed at every visit with Spectralis (Heidelberg Engineering, Heidelberg, Germany) using a 20° × 20° volume scan of 25 B-scans with automatic real-time tracking and the follow-up protocol to ensure scan registration across visits. Scans with a quality score < 15 dB were repeated. Segmentation was reviewed on every scan and manually corrected where required. Dry-macula grading, ellipsoid zone (EZ) disruption, external limiting membrane (ELM) discontinuity, and disorganization of retinal inner layers (DRIL) were graded independently by two masked readers who were not the treating clinicians; a third masked reader adjudicated disagreements. Agreement was substantial for dry macula (κ = 0.82; 95% CI, 0.74–0.90) and for EZ disruption (κ = 0.79; 95% CI, 0.70–0.88) on a random subset of 50 scans.

Measurement repeatability. Because the central claim of this study concerns the comparison of a difference against measurement error, we quantified repeatability prospectively in a random subsample of 60 patients who underwent duplicate same-day BCVA and OCT assessment. Intraclass correlation coefficients (ICC, two-way random, absolute agreement) and coefficients of repeatability (CoR = 1.96 × within-subject SD) were computed, and the signal-to-noise ratio was defined as the observed between-group difference divided by the CoR (Table 1).

Cataract was graded with LOCS III at baseline and year 5. Baseline lens status (phakic/pseudophakic) was recorded; cataract-surgery rates are reported with phakic eyes as the denominator.

### 2.5. Propensity-Score Matching

Propensity scores were estimated by multivariable logistic regression with regimen as the dependent variable and 14 baseline covariates: age, sex, diabetes type, diabetes duration, HbA1c, baseline BCVA, baseline CST, baseline lens status, diabetic retinopathy severity (moderate NPDR/severe NPDR/PDR), prior focal or grid laser, anti-VEGF agent, hypertension, hyperlipidemia, and renal disease. Matching was 1:1 nearest-neighbor without replacement, with a caliper of 0.2 SD of the logit of the propensity score.

Balance was assessed by absolute standardized mean differences (SMD < 0.10) and variance ratios (0.80–1.25); common support was assessed graphically (Figure 1B,C). Caliper widths of 0.1 and 0.3 SD were examined as sensitivity analyses. Multiple imputation (mice, 20 imputations) preceded matching, and 1:1 propensity-score matching was performed separately within each of the 20 imputed datasets (MatchIt); treatment-effect estimates from the 20 resulting matched-and-analyzed replicates were then combined using Rubin’s rules (mice::pool()). Because the matched individuals differed slightly across imputations, descriptive statistics that require integer denominators baseline characteristics (Table 1), event counts, and proportions are reported from a single pre-designated reference imputed dataset (the first imputation) after matching, in order to provide interpretable and internally consistent denominators. All inferential estimates (regression coefficients, hazard and risk ratios, confidence intervals, and *p* values) were pooled across all 20 imputed datasets using Rubin’s rules. To characterize the variability introduced by multiple imputation, we examined the distribution of matched sample sizes and balance diagnostics across all 20 imputed datasets. The number of successfully matched pairs ranged from 395 to 400 per arm. The maximum absolute standardized mean difference across the 14 baseline covariates ranged from 0.02 to 0.09 (median, 0.06), and all variance ratios remained within the prespecified 0.80–1.25 range across imputations. The exact denominators, event counts, and balance measures presented in Table 1 and in the descriptive text of Section 3.1 are therefore those of the pre-designated reference imputed dataset (the first imputation), as specified in the analysis plan.

### 2.6. Statistical Analysis

Analyses were conducted in R 4.4.1 (packages MatchIt 4.5.5, nlme 3.1-164, survival 3.7-0, WeightIt 1.2, mice 3.16, TOSTER 0.8, segmented 2.1, EValue 4.1.3) and cross-checked in Python 3.11 (statsmodels 0.14). Analysis code is deposited with the dataset.

Distributional assumptions were assessed graphically rather than by significance testing, because the Shapiro–Wilk test is uninformative at *n* = 400 per arm. Continuous variables are summarized as mean ± SD and compared with two-sample t-tests using cluster-robust standard errors accounting for the matched-pair structure; skewed variables are summarized as median [IQR] and compared with Wilcoxon signed-rank tests on matched pairs. Categorical variables were compared with McNemar tests on matched pairs. The primary outcome was tested without multiplicity adjustment; all secondary outcomes were adjusted by the Benjamini–Hochberg procedure at q = 0.05, and both raw and adjusted *p* values are reported in Table 2.

Absence of a clinically meaningful difference. We prespecified a ±5-ETDRS-letter margin, corresponding to one ETDRS line and to the conventional threshold for clinical meaningfulness in DME. TOST was performed at α = 0.05; the corresponding decision interval is the 90% CI, which is reported alongside the 95% CI. Because a 90% and a two-sided 95% interval are not interchangeable, both are given. We additionally report the smallest margin at which the TOST criterion is still met, and we repeat the analysis at a stricter ±4-letter margin. With 400 patients per arm and an observed SD of approximately 14.6 letters, the study had 99.9% power for the TOST procedure under a true difference of zero, and 80% power to detect a superiority difference of 2.8 letters.

Important methodological note. Residual confounding and informative attrition may bias observational comparisons in either direction, depending on the structure of the unmeasured factors and on the reasons for dropout; they do not systematically guarantee attenuation toward the null. Measurement error likewise does not necessarily produce a predictable or uniform attenuation of a treatment contrast in this setting. Accordingly, a finding of “no meaningful difference” in an observational study cannot be assumed to be conservative. TOST performed on non-randomized data cannot establish equivalence in the sense that a randomized equivalence trial can. Our claim is therefore restricted to the absence of a detectable clinically meaningful difference under the stated assumptions.

Longitudinal models. BCVA and CST trajectories were modeled with linear mixed-effects models containing a random intercept for a patient nested within a matched pair, categorical time (baseline and years 1–5), regimen, the regimen × time interaction, anti-VEGF agent, agent × regimen and agent × time interactions, and baseline covariates. An unstructured residual correlation structure (corSymm) was fitted for the repeated annual measurements using nlme::lme(); the random-effects structure was deliberately restricted to intercepts because a random slope for categorical time is not identifiable with six levels. Residual variances were assumed homogeneous across visits; no variance function was specified, so the fitted structure is an unstructured residual correlation matrix with a common residual variance rather than a fully unstructured residual covariance matrix. Denominator degrees of freedom were determined by the containment method, the default implemented in nlme::lme(). Residual and Q–Q plots were inspected for each model. Model fit is reported as marginal and conditional R^2^ by the method of Nakagawa.

Anatomical–functional relationship. The association between year-5 CST reduction and year-5 BCVA change was modeled by segmented (broken-stick) regression with the breakpoint estimated from the data; the Davies test was used to test for a change in slope. A restricted cubic spline model with four knots was fitted as a check.

Time to first recurrence was analyzed with Kaplan–Meier estimates and Cox models. Because recurrences can only be detected at visits and the two regimens have systematically different visit intervals, the primary time-to-event analysis is misspecified as right-censored data; we therefore also fitted an interval-censored proportional hazards model with the observation interval defined by consecutive attended visits. The proportional hazards assumption was tested with scaled Schoenfeld residuals.

Time to cataract surgery was analyzed with Kaplan–Meier estimates and Cox proportional hazards models in the 703 eyes that were phakic at baseline within the matched cohort (T&E, 353; PRN, 350); eyes pseudophakic at baseline were never at risk and were excluded. The event was the first cataract extraction performed during follow-up, identified from the surgical record; time zero was the date of the first anti-VEGF injection; and observations were censored at the last attended visit, at death, or at the 60-month cut-off, whichever occurred first. Because the phakic subset of the matched sample varied slightly between imputations, the event counts and denominators quoted in the Results are those of the pre-designated reference imputed dataset, whereas all model-based estimates were pooled across all 20 imputations using Rubin’s rules on the log-hazard scale. Two Cox models were fitted: an unadjusted model containing regimen alone, and a model adjusted for the 13 baseline covariates that varied within the phakic population (lens status being constant by definition, because the at-risk population was phakic). Both are reported so that the contribution of covariate adjustment is transparent. Because the cohort was defined by completion of 60 months of follow-up, censoring before the 60-month cut-off could arise only through death; the Kaplan–Meier estimate at five years therefore lies close to the crude proportion. The proportional hazards assumption was assessed with scaled Schoenfeld residuals.

Ascertainment bias. Monitoring frequency is a consequence of regimen and therefore lies on the causal pathway; conditioning on it would induce over-adjustment bias. Rather than adjusting for it, we (a) restricted a supporting analysis to recurrences detected at the annual comprehensive assessment common to both regimens, and (b) performed a quantitative bias analysis over plausible ranges of detection probability.

**Figure 2:**
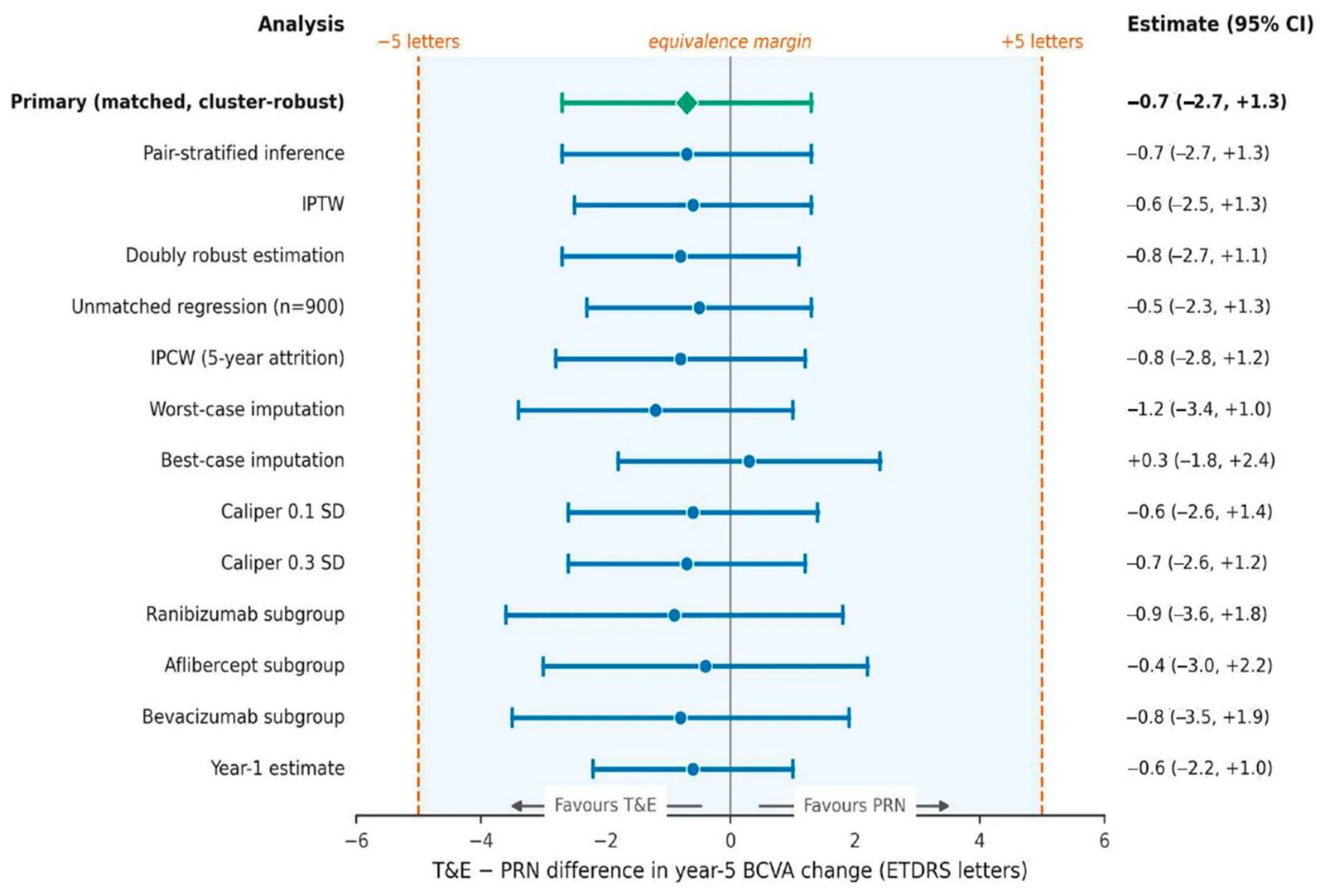
Sensitivity analyses of the primary visual estimate. Forest plot of the year-5 T&E − PRN difference in BCVA change across 14 representative analyses (of 17 prespecified). Every point estimate lay within ±1.2 letters of the primary result, and every 95% CI lay inside the ±5-letter equivalence margin (shaded). The primary estimate is shown as a diamond.

Missing data, attrition, and unmeasured confounding. Missingness among matched patients was limited to HbA1c (8.2%), diabetes duration (5.1%), and systemic comorbidities (3.5%); 20 imputations were generated by chained equations. To address the survivor design we applied inverse probability of censoring weighting using baseline predictors of non-completion (HbA1c, documented non-adherence, diabetes duration, distance from the center, and diabetic retinopathy severity), and additionally reported best-case and worst-case imputation for the 383 non-completers. Sensitivity to unmeasured confounding was quantified with E-values. Additional sensitivity analyses comprised inverse probability of treatment weighting, doubly robust augmented estimation, unmatched multivariable regression on all 900 eligible patients, alternative calipers, matched-pair stratification, complete-case analysis, exclusion of cataract-surgery patients, exclusion of agent switchers, and agent-specific subgroups (Figure 2).

All tests were two-sided with α = 0.05. This study was not pre-registered; the analysis plan was fixed before the outcome dataset was unblinded to the analyst and is archived with the data.

## 3. Results

### 3.1. Patient Disposition and Baseline Comparability

Of 1283 patients screened, 383 (29.9%) did not complete 60 months of follow-up, leaving 900 eligible patients: 473 (52.6%) treated with T&E and 427 (47.4%) with PRN. One-to-one matching within a 0.2 SD caliper yielded 400 pairs; 73 T&E and 27 PRN patients fell outside the common support and were excluded, giving a final cohort of 800 eyes of 800 patients (Figure 1). The disposition shown in Figure 1 is that of the pre-designated reference imputed dataset; across the 20 imputed datasets the number of matched pairs ranged from 395 to 400.

The 383 excluded patients were unable to complete five years of follow-up for various reasons: treatment discontinuation or documented non-adherence (*n* = 156, 12.2%), incomplete records (*n* = 89, 6.9%), loss to follow-up (*n* = 62, 4.8%), migration out of the catchment area (*n* = 42, 3.3%), death from causes unrelated to therapy (*n* = 23, 1.8%), and cataract surgery precluding accurate visual assessment (*n* = 11, 0.9%). These patients were not excluded because of worse glycemic control; rather, they were unable to maintain treatment and follow-up. The distribution of reasons for non-completion did not differ between regimens (*p* = 0.62).

After matching, all 14 covariates were balanced (all |SMD| ≤ 0.07; all variance ratios 0.88–1.14; Table 1, Figure 1B) in the reference imputed dataset, with the corresponding ranges across all 20 imputations reported in Section 2.5, and propensity-score distributions overlapped substantially (Figure 1C). The three baseline characteristics that were recorded but not included in the propensity model, subretinal fluid, DRIL, and EZ disruption, were also well balanced (all |SMD| ≤ 0.02), which is expected given their association with the matched covariates, in particular baseline BCVA and CST. Mean age was 62.8 ± 9.2 years (T&E) and 63.4 ± 9.0 years (PRN); baseline BCVA was 59.1 ± 15.3 and 59.8 ± 14.8 letters; baseline CST was 425.9 ± 102.5 and 419.8 ± 98.7 µm. The counts and proportions reported throughout the Results are those of the pre-designated reference-imputed dataset after matching, whereas all effect estimates, confidence intervals and *p* values are pooled across all 20 imputations.

### 3.2. Measurement Repeatability

Same-day duplicate assessment in 60 patients gave an ICC of 0.962 (95% CI, 0.938–0.977) for BCVA with a coefficient of repeatability of 4.8 letters, and an ICC of 0.981 (95% CI, 0.969–0.989) for CST with a coefficient of repeatability of 12.4 µm (Table 1). The observed year-5 between-group difference in BCVA (0.7 letters) was therefore 0.15 times the measurement noise of the instrument, whereas the CST difference (58.1 µm) was 4.7 times its measurement noise. This asymmetry is central to the interpretation of the results that follow: the anatomical difference is measurable, and the visual difference is not.

### 3.3. Visual Outcomes

**Figure 3:**
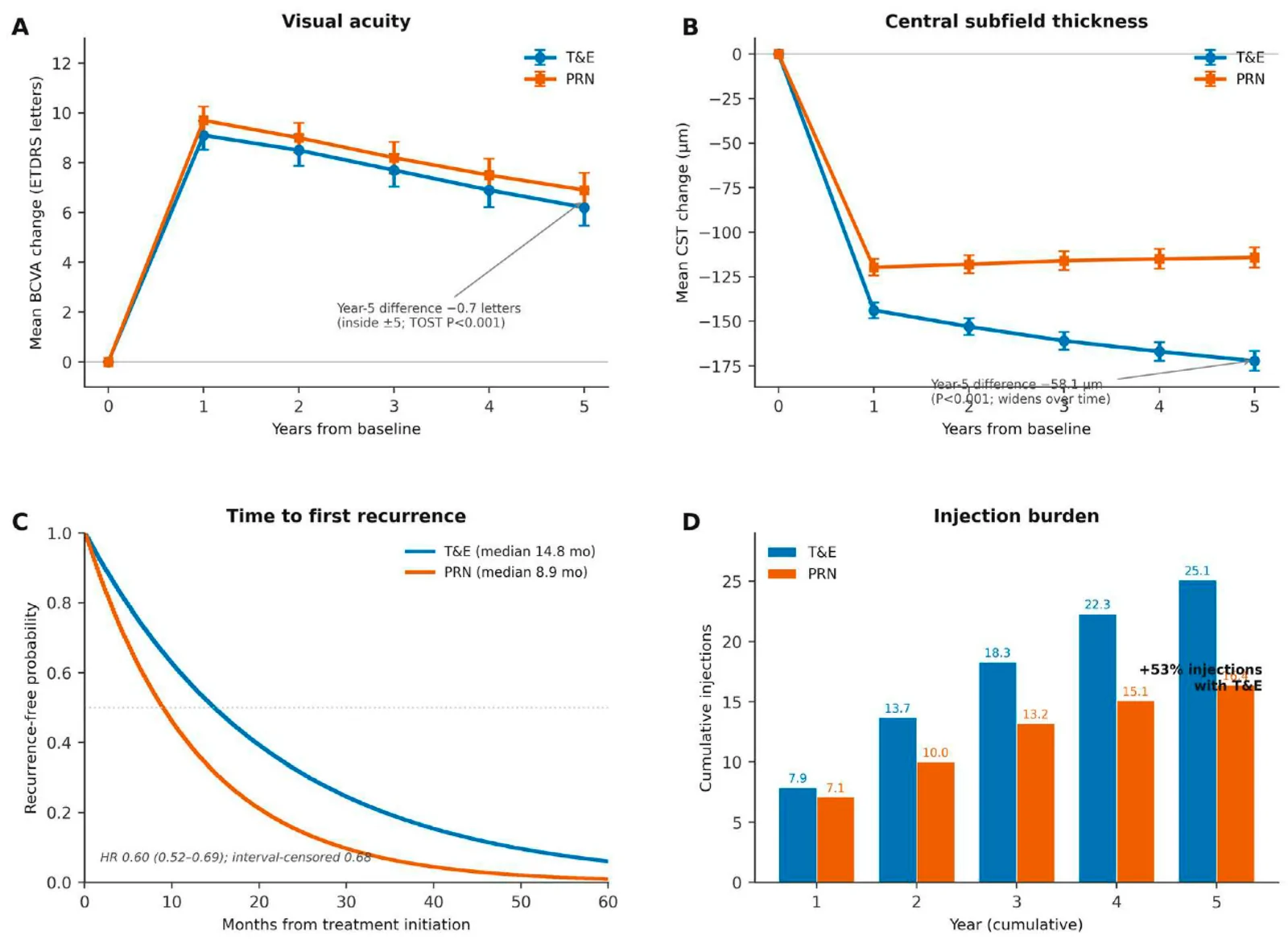
Five-year clinical trajectories. (**A**) Mean BCVA change; the year-5 difference of −0.7 letters lies well inside the prespecified ±5-letter margin (TOST *p* < 0.001). (**B**) Mean CST change; the T&E advantage widens over time to −58.1 µm at year 5 (*p* < 0.001). (**C**) Kaplan–Meier recurrence-free survival (median 14.8 vs. 8.9 months; crude HR 0.60, attenuating to 0.68 under interval-censored modeling). (**D**) Cumulative injection burden (25.1 vs. 16.4; +53% with T&E). Error bars denote standard errors. Grey lines are reference guides: the horizontal line in (**A**,**B**) marks zero change from baseline, the diagonal lines are annotation leaders, and the dotted line in (**C**) marks 0.50 recurrence-free probability.

Both regimens improved vision substantially and both lost part of the early gain (Figure 3A). At year 1, BCVA improved by 9.1 ± 11.6 letters (T&E) and 9.7 ± 11.2 letters (PRN); at year 5 by 6.2 ± 14.6 and 6.9 ± 14.0 letters (all within-group *p* < 0.001).

The year-5 between-group difference was −0.7 letters (90% CI, −2.4 to 1.0; 95% CI, −2.7 to 1.3; *p* = 0.49). The 90% CI, which is the decision interval for TOST at α = 0.05, lay entirely within the prespecified ±5-letter margin (TOST *p* < 0.001), as did the more conservative 95% CI. The criterion was still met at a stricter ±4-letter margin (TOST *p* < 0.001) and, indeed, at any margin wider than 2.4 letters. At year 1 the difference was −0.6 letters (95% CI, −2.2 to 1.0; *p* = 0.46). The regimen × time interaction for BCVA was not significant (*p* = 0.71).

Categorical outcomes were likewise similar: ≥15-letter gain in 91 eyes (22.8%) with T&E versus 98 (24.5%) with PRN (difference −1.7%; 95% CI, −7.6 to 4.2; *p* = 0.56) and ≥15-letter loss in 58 (14.5%) versus 53 (13.3%) (difference 1.2%; 95% CI, −3.5 to 6.0; *p* = 0.61).

### 3.4. Anatomical Outcomes

Anatomical outcomes diverged, and the divergence widened over time (regimen × time interaction *p* < 0.001; Figure 3B). CST reduction at year 1 was −143.8 ± 88.4 µm with T&E versus −119.7 ± 94.1 µm with PRN (difference −24.1 µm; 95% CI, −36.8 to −11.4; *p* < 0.001), and at year 5 was −172.3 ± 111.0 versus −114.2 ± 113.6 µm (difference −58.1 µm; 95% CI, −73.7 to −42.5; *p* < 0.001; Cohen d = 0.52).

Complete fluid resolution at year 5 was achieved in 295 eyes (73.8%) with T&E versus 238 (59.5%) with PRN (difference 14.3%; 95% CI, 7.8 to 20.7; risk ratio 1.24; 95% CI, 1.12 to 1.37; *p* < 0.001). The corresponding E-value was 1.78 (1.49 for the CI limit): an unmeasured confounder would have to be associated with both regimen assignment and dry macula by a risk ratio of at least 1.78, above and beyond the 14 matched covariates, to explain this association away.

### 3.5. The Anatomical–Functional Relationship

**Figure 4:**
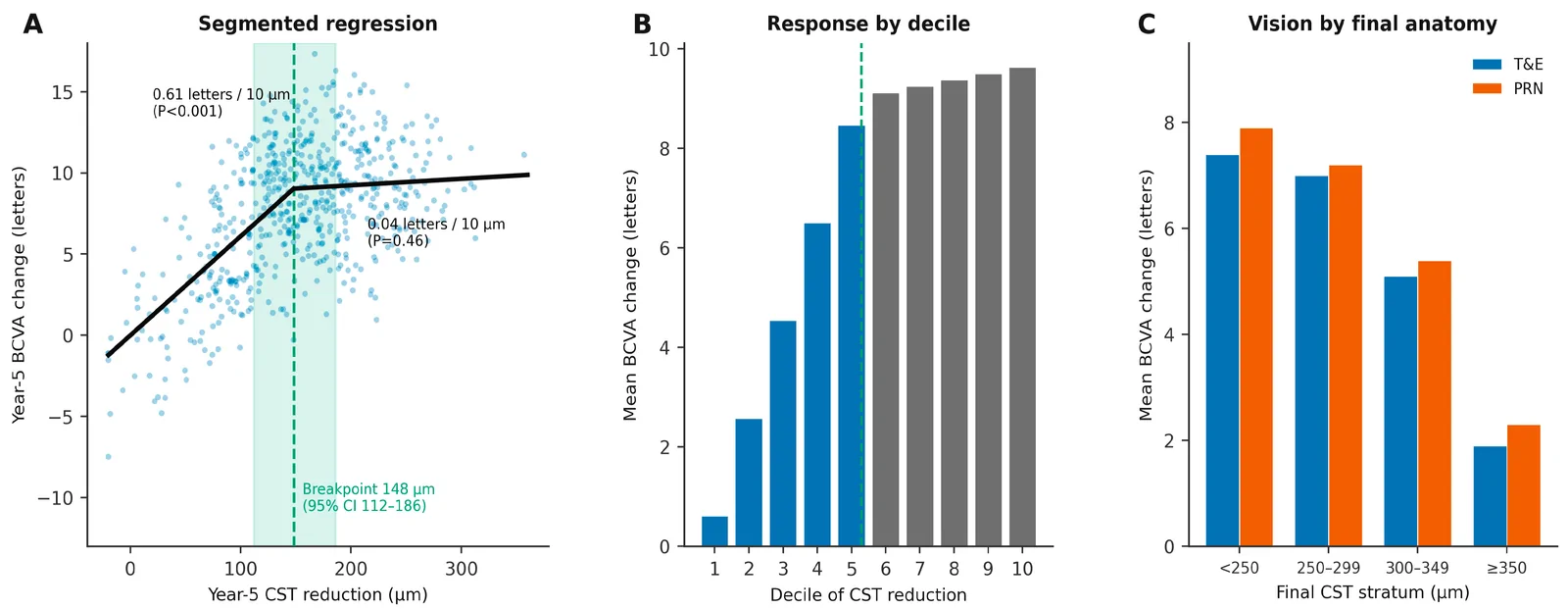
Anatomical–functional dissociation. (**A**) Segmented regression of year-5 BCVA change on year-5 CST reduction, with a breakpoint at 148 µm (95% CI, 112–186; green band). Below the breakpoint the slope is 0.61 letters/10 µm (*p* < 0.001); above it, 0.04 letters/10 µm (*p* = 0.46). (**B**) Mean BCVA change by decile of anatomical response, showing a plateau above the breakpoint. (**C**) Year-5 BCVA change by final CST stratum and regimen: final anatomy predicts vision (*p* for trend < 0.001) but regimen does not differ within any stratum.

Segmented regression of year-5 BCVA change on year-5 CST reduction identified a breakpoint at 148 µm (95% CI, 112–186; Davies test *p* < 0.001; Figure 4A). Below the breakpoint, each additional 10 µm of CST reduction was associated with a gain of 0.61 letters (95% CI, 0.41 to 0.81; *p* < 0.001). Above it, the slope was 0.04 letters per 10 µm (95% CI, −0.07 to 0.15; *p* = 0.46) statistically and clinically indistinguishable from zero. A four-knot restricted cubic spline reproduced the same plateau.

Consistent with this, mean year-5 BCVA change fell monotonically across categories of final CST (<250 µm: +7.6 letters; 250–299 µm: +7.1; 300–349 µm: +5.2; ≥350 µm: +2.1; *p* for trend < 0.001), but within every stratum the two regimens were indistinguishable (all *p* ≥ 0.30; Figure 4C). In this cohort, therefore, final anatomical status was associated with year-5 vision and regimen was associated with final anatomical status, but once final anatomical status was accounted for, regimen was not associated with any further difference in vision. This pattern is observational and does not establish that the anatomical difference is causally inert.

### 3.6. Treatment Burden, Monitoring, and Recurrence

Cumulative injections over five years were 25.1 ± 6.5 with T&E versus 16.4 ± 7.3 with PRN (difference 8.7; 95% CI, 7.7 to 9.7; *p* < 0.001), a 53% relative increase, with the gap widening annually (Figure 3D). Twenty or more injections were required by 330 T&E eyes (82.5%) versus 154 PRN eyes (38.5%) (risk ratio 2.14; 95% CI, 1.88 to 2.44), and ten or fewer by 16 (4.0%) versus 91 (22.8%) (risk ratio 0.18; 95% CI, 0.11 to 0.29).

The burden traded in the opposite direction for monitoring: injection-free visits numbered 14.6 ± 6.2 with T&E versus 28.4 ± 8.7 with PRN (difference −13.8; 95% CI, −14.9 to −12.7; *p* < 0.001). Total attended visits were 39.7 ± 7.4 versus 44.8 ± 9.1 (*p* < 0.001). Across the cohort, 16,600 injections were administered.

Recurrences over five years numbered 2.2 ± 1.5 with T&E versus 3.9 ± 2.0 with PRN (difference −1.7; 95% CI, −1.9 to −1.5; incidence rate ratio 0.56; 95% CI, 0.52 to 0.61; *p* < 0.001). Median time to first recurrence was 14.8 months (IQR, 8.2–22.4) with T&E versus 8.9 months (IQR, 4.6–15.2) with PRN (log-rank *p* < 0.001; hazard ratio 0.60; 95% CI, 0.52 to 0.69; Figure 3C). The proportional hazards assumption was satisfied (global Schoenfeld *p* = 0.21).

These recurrence estimates are, however, ascertainment-dependent. Under interval-censored modeling with the observation window defined by consecutive attended visits, the hazard ratio attenuated to 0.68 (95% CI, 0.57 to 0.81). Restricting to recurrences detected at the annual comprehensive assessment attended by both arms, recurrence was documented in 245 T&E eyes (61.3%) versus 299 PRN eyes (74.8%) (risk ratio 0.82; 95% CI, 0.74 to 0.91; *p* < 0.001). Quantitative bias analysis indicated that the direction of effect was preserved across the full plausible range of differential detection probability, but that the magnitude of the crude estimate is inflated by roughly 20–30%.

### 3.7. Adjunctive Therapy, Cataract, and Safety

Adjunctive and rescue interventions were numerically less frequent with T&E but no comparison reached significance after adjustment: focal or grid laser 24 (6.0%) versus 38 (9.5%) (*p* = 0.07), panretinal photocoagulation for retinopathy progression 42 (10.5%) versus 58 (14.5%) (*p* = 0.09), dexamethasone implant rescue 18 (4.5%) versus 29 (7.3%) (*p* = 0.11), and pars plana vitrectomy 7 (1.8%) versus 11 (2.8%) (*p* = 0.34) (Table 3).

Cataract surgery. Among the 703 eyes phakic at baseline, the 5-year cumulative incidence by Kaplan–Meier estimation was 9.6% (95% CI, 6.8–13.2) in the T&E arm and 9.1% (95% CI, 6.3–12.7) in the PRN arm (34 of 353 and 32 of 350 eyes, respectively). With 66 cataract events in total, the unadjusted Cox hazard ratio for T&E versus PRN, fitted in the same 20 matched replicates and pooled by Rubin’s rules, was 1.04 (95% CI, 0.64–1.69), consistent in direction with the slightly higher crude proportion in the T&E arm. After adjustment for the 13 baseline covariates that varied within the phakic subgroup (age, sex, diabetes type, diabetes duration, HbA1c, baseline BCVA, baseline CST, diabetic retinopathy severity, prior laser, anti-VEGF agent, hypertension, hyperlipidemia, and renal disease), the pooled hazard ratio was 0.97 (95% CI, 0.60–1.60; *p* = 0.90). The direction of the point estimate shifted marginally with adjustment, reflecting residual imbalance in covariates associated with cataract development; this shift is small relative to the imprecision of both estimates, whose confidence intervals overlap substantially and are compatible with no difference between regimens. The proportional hazards assumption was satisfied (Schoenfeld *p* = 0.80).

Among the 637 eyes still phakic at year 5, LOCS III scores did not differ between regimens in any subscale (all adjusted *p* > 0.05). Contributing factors to the surgical rate include a mean surgical waiting time of 14.2 ± 6.8 months at our institution, a practice of deferring surgery until DME was controlled, probable under-ascertainment of surgery performed elsewhere, and limited access in a rural catchment. This limits generalizability to systems with prompt surgical access and may also mean that lens-related acuity loss is differentially unresolved in our year-5 measurements.

Ocular adverse events were comparable: subconjunctival hemorrhage 170 (42.5%) versus 155 (38.8%) (*p* = 0.28), transient ocular discomfort 114 (28.5%) versus 102 (25.5%) (*p* = 0.34), and intraocular pressure elevation requiring treatment 32 (8.0%) versus 26 (6.5%) (*p* = 0.41). No case of endophthalmitis, rhegmatogenous retinal detachment, or severe intraocular inflammation was recorded in 16,600 injections, each reported separately in Table 3; because each of these three outcomes had zero observed events over the same denominator, the exact one-sided 97.5% upper confidence bound is 0.018% (1 in 5533) for each, which does not exclude rates consistent with published series, and zero observed events should not be read as evidence of superior sterility or surgical safety. APTC-defined arterial thromboembolic events occurred in 21 (5.3%) versus 19 (4.8%) patients (*p* = 0.75); by design the cohort excludes patients who died during follow-up, so systemic safety cannot be assessed properly here.

### 3.8. Multivariable and Mixed-Effects Models

In the mixed-effects model for BCVA, the PRN arm gained 9.7 letters (95% CI, 8.6 to 10.8) at year 1 and 6.9 letters (95% CI, 5.5 to 8.3) at year 5. The T&E–PRN contrast was −0.6 letters (95% CI, −2.2 to 1.0) at year 1 and −0.7 letters (95% CI, −2.7 to 1.3) at year 5, with a non-significant overall regimen × time interaction (*p* = 0.71). Marginal and conditional R^2^ were 0.31 and 0.72.

For CST, the PRN arm changed by −119.7 µm (95% CI, −128.9 to −110.5) at year 1 and −114.2 µm (95% CI, −125.3 to −103.1) at year 5, with T&E–PRN contrasts of −24.1 µm (95% CI, −36.8 to −11.4) and −58.1 µm (95% CI, −73.7 to −42.5), and a significant regimen × time interaction (*p* < 0.001). Marginal and conditional R^2^ were 0.38 and 0.79.

Anti-VEGF agent effects were small and imprecise. Relative to ranibizumab, aflibercept was associated with +1.2 letters at year 5 (95% CI, −0.9 to 3.3; *p* = 0.26) and −14.8 µm of CST (95% CI, −31.9 to 2.3; *p* = 0.09); bevacizumab differed from ranibizumab on neither outcome. No agent × regimen interaction approached significance for either outcome (all *p* ≥ 0.77), and the agent-specific estimates of the regimen contrast all lay within the ±5-letter margin, although with wide intervals reflecting subgroup sample sizes of 260–276 (Figure 2).

Multivariable modeling of year-5 BCVA identified baseline BCVA (β = 0.48 letters per letter; 95% CI, 0.40 to 0.56; *p* < 0.001), baseline EZ disruption (β = −4.2 letters; 95% CI, −6.6 to −1.8; *p* < 0.001), PDR versus moderate NPDR (β = −3.1 letters; 95% CI, −5.4 to −0.8; *p* = 0.008), baseline CST (β = −1.5 letters per 100 µm; 95% CI, −2.1 to −0.9; *p* < 0.001), age (β = −1.3 letters per decade; 95% CI, −2.4 to −0.2; *p* = 0.02), and HbA1c (β = −0.9 letters per 1%; 95% CI, −1.7 to −0.1; *p* = 0.03) as independent predictors (R^2^ = 0.42). Neither regimen (β = −0.6; 95% CI, −2.5 to 1.3; *p* = 0.53) nor cumulative injection number (β = 0.2 per 5 injections; 95% CI, −0.5 to 0.9; *p* = 0.58) predicted year-5 vision (Table 4). All variance inflation factors were below 2.5.

### 3.9. Sensitivity Analyses

The year-5 visual estimate was stable across all 17 prespecified sensitivity analyses (Figure 2). Matched-pair cluster-robust and pair-stratified inference reproduced the primary estimate exactly. Inverse probability of treatment weighting gave −0.6 letters (95% CI, −2.5 to 1.3), doubly robust estimation −0.8 (−2.7 to 1.1), and unmatched multivariable regression on all 900 eligible patients −0.5 (−2.3 to 1.3). IPCW for five-year attrition gave −0.8 letters (95% CI, −2.8 to 1.2). Worst-case imputation for the 383 non-completers gave −1.2 letters (95% CI, −3.4 to 1.0) and best-case imputation +0.3 (−1.8 to 2.4); both remain inside the equivalence margin. Every point estimate lay within ±1.2 letters of the primary result, and every interval lay inside the ±5-letter margin.

## 4. Discussion

In 400 propensity-score–matched pairs followed for five years, a treat-and-extend regimen produced substantially better anatomical control than pro re nata dosing 58 µm more central thickness reduction, 14 percentage points more complete fluid resolution, and roughly a third fewer recurrences but no corresponding advantage in visual acuity was detected. The visual difference at five years was 0.7 letters, which is less than one-sixth of the coefficient of repeatability of the acuity measurement itself. The reason emerges from the segmented regression: the relationship between anatomical drying and visual gain is not linear but saturating, with a plateau beginning at approximately 150 µm of CST reduction. In this cohort, the incremental drying achieved with T&E was located predominantly beyond that plateau, in a region where further thickness reduction was not associated with additional detectable visual gain. This should not be read as evidence that further anatomical improvement is without clinical value for all patients.

This reframes the comparison. The relevant question is not whether T&E achieves better anatomical control it plainly does but whether the additional anatomical gain lies in a range in which it is accompanied by measurable visual benefit. In this cohort, no such benefit was detected.

Our five-year gains (+6.2 and +6.9 letters) sit between the Protocol T extension cohort (+7.4 letters at five years in patients recalled after protocol-driven treatment) and PRN-based real-world series reporting approximately +5 letters, and above registry cohorts [13]. The most plausible explanation is treatment intensity rather than regimen: our PRN patients received 7.1 injections in the first year, more than most reported real-world PRN cohorts, and 16.4 over five years. The corollary is uncomfortable but it is important that a well-executed PRN regimen and a well-executed T&E regimen appear to converge functionally, while a poorly executed regimen of either kind does not [16].

The dissociation we describe echoes findings outside DME. The FLUID study showed that tolerating subretinal fluid in neovascular AMD did not compromise two-year vision and post hoc DRCR analyses have repeatedly failed to find a tight coupling between residual thickness and letter score [17,18]. Our contribution is to estimate the location of the inflection rather than to assert its existence, and to show that it falls where regimen-driven differences in drying actually occur.

Several mechanisms plausibly generate the plateau. Photoreceptor and outer-retinal damage accumulates early and is not reversed by later drying; EZ disruption was present in 36.4% and ELM discontinuity in 26.9% of eyes at year 5, and baseline EZ disruption was one of the strongest independent predictors of year-5 acuity in our model. Macular ischemia progresses independently of fluid control and was not assessable here without OCT angiography [19]. Chronic Müller-cell and inner-retinal reorganization, reflected in DRIL, may impose a functional ceiling regardless of thickness [20]. Finally, a cumulative burden of 25 injections is not biologically free even when no discrete adverse event is recorded.

T&E halved the recurrence rate on crude analysis, but PRN patients were examined nearly twice as often, and a recurrence that is never looked for is never counted. We deliberately did not adjust for monitoring frequency, because monitoring frequency is a consequence of regimen and lies on the causal pathway; conditioning on it would introduce over-adjustment bias, a common error in this literature. Interval-censored modeling and restriction to the annual assessment common to both arms both attenuated the effect (hazard ratio 0.60 → 0.68; risk ratio 0.82 at the common assessment). A genuine biological difference survives, but the crude estimate overstates it by roughly a quarter, and reports that adjust for visit frequency and claim an undiminished effect should be read skeptically.

Two practical conclusions follow. First, where drug cost dominates as it does with aflibercept and ranibizumab, and as it does not with bevacizumab, the 53% injection premium of T&E was associated with additional anatomical drying but with no detectable additional visual gain in this adherent cohort. Second, where travel and attendance cost dominates, as in the geographically dispersed population served by our center, T&E’s halving of monitoring-only visits is the operative advantage and may be decisive. These are opposite conclusions in different health systems, and both are supported by the same dataset. However, formal cost-utility analysis was not performed in this study; cost-related conclusions are hypothesis-generating and should be confirmed with formal economic evaluation.

The advent of dual angiopoietin-2/VEGF-A inhibition sharpens rather than dissolves this question. Faricimab achieves extended intervals with maintained anatomical control in a substantial majority of patients, which will push more practice toward proactive extended dosing [21,22,23]. Our data suggest that the value of that shift should be measured in visits avoided and disease stability preserved, not in letters gained, and that trials of newer agents should prespecify anatomical and functional endpoints with an explicit hypothesis about the relationship between them rather than assuming one.

### 4.1. Limitations

First and most importantly, this was a survivor cohort that conditioned analyses on completion of 60 months of follow-up. Consequently, 383 patients (29.9%) who discontinued treatment, were lost to follow-up, migrated, died, or had incomplete records were excluded. These patients were excluded because they could not complete follow-up, not because of worse glycemic control. Therefore, our findings apply only to adherent patients who completed a structured treatment program and should not be generalized to non-adherent or high-risk populations, in whom the proactive nature of T&E may provide greater benefit. Although inverse probability of censoring weighting and best-/worst-case imputations yielded similar estimates, these approaches rely on unverifiable assumptions and do not overcome survivor selection; accordingly, our estimand remains the treatment effect among patients who completed five years rather than an intention-to-treat effect.

Second, treatment allocation was not randomized. Despite extensive propensity-score matching and multiple sensitivity analyses, residual confounding from unmeasured factors, including physician judgment, socioeconomic status, and cognitive function, cannot be excluded. Moreover, residual confounding and informative attrition may bias the estimated treatment contrast in either direction rather than reliably toward the null, and TOST performed in a non-randomized setting cannot establish equivalence in the same manner as a randomized equivalence trial.

Third, time-varying systemic factors, such as longitudinal HbA1c, blood pressure, and renal function, were incompletely recorded, precluding marginal structural modeling. Fourth, recurrence detection differed between regimens because of different monitoring schedules, although interval-censored analyses suggested that the primary conclusions were robust. Fifth, interpretation of cataract outcomes requires caution because the survivor design, delayed access to cataract surgery, and possible under-ascertainment of surgery performed elsewhere may have influenced year-5 visual acuity estimates. In addition, the adjusted Cox model contained 13 covariates for 66 events, corresponding to approximately five events per variable; the adjusted hazard ratio should therefore be regarded as imprecise and potentially subject to overfitting, although its close agreement with the unadjusted estimate argues against a material distortion.

Finally, the single-center setting, involvement of only two treating physicians, and use of a single OCT platform limit external validity. OCT angiography was unavailable, preventing longitudinal assessment of macular ischemia, while outer retinal integrity was graded only at year 5. Patient-reported outcomes and formal cost-effectiveness analyses were not performed despite the study’s emphasis on treatment burden, no meaningful safety conclusions can be drawn from the absence of endophthalmitis events, and the study was not prospectively registered. Although the analysis plan was fixed and archived before the outcome dataset was unblinded to the analyst, it was not lodged on a public registry; formal prospective registration would have provided independent, time-stamped verification of the pre-specified plan. We are not aware of any deviations from the archived plan, but a registered study would allow this to be confirmed with greater certainty by readers and editors.

### 4.2. Clinical Implications

For adherent patients able to sustain five years of continuous therapy and follow-up, these findings suggest that clinicians may reasonably select between T&E and PRN on practical grounds, including injection burden, monitoring capacity, travel distance, and patient preference, without an expected sacrifice in five-year visual outcome. In this cohort, no additional visual gain was detected beyond the exploratory 148-µm breakpoint of CST reduction; accordingly, escalating treatment intensity solely to pursue near-complete anatomical drying beyond this exploratory point was not associated with additional visual gain in this cohort, although anatomical control could still carry value for outcomes not captured by BCVA, such as recurrence risk and monitoring burden. These implications are specific to patients who can maintain long-term follow-up and should not guide treatment-intensity decisions in non-adherent or high-risk populations.

## 5. Conclusions

Among patients with center-involving DME who sustained five years of continuous anti-VEGF therapy, treat-and-extend achieved markedly superior anatomical control without any detectable visual advantage over pro re nata dosing, at the cost of 53% more injections and the benefit of half as many monitoring-only visits. A plausible explanation is a saturating anatomical–functional relationship: beyond an exploratory, cohort-specific breakpoint of approximately 150 µm of central thickness reduction, further drying was not associated with additional measurable visual gain in this cohort. As an observational association from change-from-baseline analyses, this should not be interpreted as evidence that anatomical improvement beyond this point is clinically inconsequential for all patients. Baseline visual acuity, outer-retinal integrity, and retinopathy severity not regimen determined five-year vision. Regimen selection should therefore be individualized on the basis of injection burden, monitoring capacity, geography, and patient preference. Formal cost-utility analysis was not performed in this study. The 5-year cumulative incidence of cataract surgery was approximately 9–10% (34/353 in the T&E arm and 32/350 in the PRN arm), lower than the 30–50% rates reported in some Western cohorts, possibly reflecting differences in baseline lens status, surgical threshold, or duration of follow-up documentation in this population. Because the cohort was defined by five-year survival and excludes patients who could not maintain follow-up, these conclusions describe adherent patients only; they should not be interpreted as evidence against the use of T&E in non-adherent or high-risk populations, where T&E may offer greater advantage. A randomized equivalence trial with cost-utility analysis and patient-reported outcomes in an unselected population remains necessary.

## Figures and Tables

**Table 1:** Baseline Characteristics and Measurement Repeatability in the Propensity Score–Matched Cohort.

**A. Baseline Characteristics (Propensity-Score–Matched Cohort)**			
**Characteristic**	**T&E (*n* = 400)**	**PRN (*n* = 400)**	**SMD**
**Demographic**			
Age, years	62.8 ± 9.2	63.4 ± 9.0	0.07
Female sex, *n* (%)	184 (46.0)	192 (48.0)	0.04
**Systemic**			
Diabetes type 1/type 2, *n*	48/352	52/348	0.03
Diabetes duration, years	12.4 ± 6.8	12.1 ± 6.5	0.05
HbA1c, %	8.5 ± 1.5	8.6 ± 1.6	0.06
Hypertension, *n* (%)	268 (67.0)	272 (68.0)	0.02
Hyperlipidemia, *n* (%)	196 (49.0)	188 (47.0)	0.04
Renal disease, *n* (%)	112 (28.0)	116 (29.0)	0.02
**Ocular**			
BCVA, ETDRS letters	59.1 ± 15.3	59.8 ± 14.8	0.05
CST, µm	425.9 ± 102.5	419.8 ± 98.7	0.06
Subretinal fluid, *n* (%)	104 (26.0)	100 (25.0)	0.02
DRIL present, *n* (%)	132 (33.0)	128 (32.0)	0.02
EZ disruption, *n* (%)	88 (22.0)	84 (21.0)	0.02
Phakic lens, *n* (%)	353 (88.3)	350 (87.5)	0.02
Moderate NPDR, *n* (%)	168 (42.0)	176 (44.0)	0.04
Severe NPDR, *n* (%)	148 (37.0)	142 (35.5)	0.03
Proliferative DR, *n* (%)	84 (21.0)	82 (20.5)	0.01
Prior laser, *n* (%)	96 (24.0)	92 (23.0)	0.02
Anti-VEGF agent, R/A/B, *n*	124/144/132	136/132/132	0.06
**B. Measurement Repeatability (Same-Day Duplicate, *n* = 60)**			
**Measurement**	**ICC (95% CI)**	**CoR**	**Signal/Noise**
BCVA, letters	0.962 (0.938–0.977)	4.8 letters	0.15
CST, µm	0.981 (0.969–0.989)	12.4 µm	4.69
Dry macula (κ)	0.82 (0.74–0.90)	—	—

All SMD < 0.10, indicating successful balance. Matching covariates (14 total): age, sex, diabetes type, diabetes duration, HbA1c, baseline BCVA, baseline CST, baseline lens status, diabetic retinopathy severity (moderate NPDR/severe NPDR/PDR), prior laser, anti-VEGF agent, hypertension, hyperlipidemia, and renal disease. The additional rows shown (subretinal fluid, DRIL, EZ disruption) are descriptive baseline characteristics not used in matching. Values are mean ± SD or *n* (%). BCVA = best-corrected visual acuity; CST = central subfield thickness; DR = diabetic retinopathy; DRIL = disorganization of retinal inner layers; EZ = ellipsoid zone; NPDR = non-proliferative diabetic retinopathy; R/A/B = ranibizumab/aflibercept/bevacizumab; SMD = standardized mean difference. ICC = intraclass correlation coefficient; CoR = coefficient of repeatability (1.96 × within-subject SD). Signal/noise = between-group difference ÷ CoR; values > 1 indicate the difference exceeds measurement noise.

**Table 2:** Five-Year Visual and Anatomical Outcomes by Treatment Regimen.

Outcome	T&E (*n* = 400)	PRN (*n* = 400)	Difference (95% CI)	*p*
**Visual outcomes**				
BCVA change, year 1, letters	+9.1 ± 11.6	+9.7 ± 11.2	−0.6 (−2.2 to 1.0)	0.46
BCVA change, year 5, letters	+6.2 ± 14.6	+6.9 ± 14.0	−0.7 (−2.7 to 1.3)	0.49
—90% CI (TOST)			−0.7 (−2.4 to 1.0)	<0.001
≥15-letter gain, *n* (%)	91 (22.8)	98 (24.5)	−1.7% (−7.6 to 4.2)	0.56
≥15-letter loss, *n* (%)	58 (14.5)	53 (13.3)	+1.2% (−3.5 to 6.0)	0.61
**Anatomical outcomes**				
CST change, year 1, µm	−143.8 ± 88.4	−119.7 ± 94.1	−24.1 (−36.8 to −11.4)	<0.001
CST change, year 5, µm	−172.3 ± 111.0	−114.2 ± 113.6	−58.1 (−73.7 to −42.5)	<0.001
Dry macula, year 1, *n* (%)	210 (52.5)	165 (41.3)	+11.2% (+4.4 to 18.1)	0.001
Dry macula, year 5, *n* (%)	295 (73.8)	238 (59.5)	+14.3% (+7.8 to 20.7)	<0.001
**Outer retina (year 5)**				
EZ disruption, *n* (%)	143 (35.8)	148 (37.0)	−1.2% (−7.9 to 5.4)	0.72
ELM discontinuity, *n* (%)	106 (26.5)	109 (27.3)	−0.8% (−6.9 to 5.4)	0.80

Values are mean ± SD or *n* (%). Negative CST differences favor T&E. Highlighted rows indicate the primary anatomical–functional dissociation finding. ELM = external limiting membrane; EZ = ellipsoid zone; TOST = two one-sided tests.

**Table 3:** Treatment Burden, Recurrence, Cataract Surgery, and Safety Over Five Years.

Outcome	T&E (*n* = 400)	PRN (*n* = 400)	Effect (95% CI)	*p*
**Treatment burden**				
Total injections, *n*	25.1 ± 6.5	16.4 ± 7.3	+8.7 (7.7 to 9.7)	<0.001
Year 1 injections, *n*	7.9 ± 1.3	7.1 ± 1.9	+0.8 (0.6 to 1.0)	<0.001
Year 3 injections, *n*	4.6 ± 1.6	3.2 ± 1.8	+1.4 (1.2 to 1.6)	<0.001
Year 5 injections, *n*	2.8 ± 2.3	1.3 ± 2.0	+1.5 (1.2 to 1.8)	<0.001
≥20 injections, *n* (%)	330 (82.5)	154 (38.5)	RR 2.14 (1.88–2.44)	<0.001
**Monitoring**				
Monitoring-only visits, *n*	14.6 ± 6.2	28.4 ± 8.7	−13.8 (−14.9 to −12.7)	<0.001
Total visits, *n*	39.7 ± 7.4	44.8 ± 9.1	−5.1 (−6.3 to −3.9)	<0.001
Adherence, %	92.4 ± 8.1	74.7 ± 15.2	+17.7 (16.0 to 19.4)	<0.001
**Recurrence**				
Recurrences, *n* (5-year)	2.2 ± 1.5	3.9 ± 2.0	IRR 0.56 (0.52–0.61)	<0.001
Median time to first, mo	14.8 [8.2–22.4]	8.9 [4.6–15.2]	HR 0.60 (0.52–0.69)	<0.001
—interval-censored model			HR 0.68 (0.57–0.81)	<0.001
—annual common assessment	245 (61.3)	299 (74.8)	RR 0.82 (0.74–0.91)	<0.001
**Cataract (phakic at baseline)**				
5-year cumulative incidence (KM)	9.6% (6.8–13.2)	9.1% (6.3–12.7)	Adjusted HR 0.97 (0.60–1.60)	0.90
Crude proportion	34/353 (9.6%)	32/350 (9.1%)	RR 1.05 (0.67–1.67)	0.82
—unadjusted Cox model	—	—	HR 1.04 (0.64–1.69)	0.87
Safety				
Endophthalmitis, *n* (%)	0 (0)	0 (0)	—	>0.99
Retinal detachment, *n* (%)	0 (0)	0 (0)	—	>0.99
Severe intraocular inflammation, *n* (%)	0 (0)	0 (0)	—	>0.99
IOP elevation, *n* (%)	32 (8.0)	26 (6.5)	RR 1.23 (0.75–2.03)	0.41
APTC event, *n* (%)	21 (5.3)	19 (4.8)	RR 1.11 (0.61–2.01)	0.75

Values are mean ± SD, median [IQR], or *n* (%). Total injections administered: 16,600. Exact one-sided 97.5% upper bound for endophthalmitis: 0.018% (1 in 5533). APTC = Antiplatelet Trialists’ Collaboration; HR = hazard ratio; IOP = intraocular pressure; IRR = incidence rate ratio; KM = Kaplan–Meier; RR = risk ratio. Cataract event definition: first cataract extraction during follow-up; at-risk population: the 703 eyes phakic at baseline in the matched cohort (T&E, 353; PRN, 350); time zero: date of the first anti-VEGF injection; censoring at the last attended visit, death, or the 60-month cut-off, whichever occurred first. Counts are those of the reference imputed dataset; the hazard ratio, confidence interval and *p* value are pooled across the 20 imputations. Because the cohort was restricted to patients completing 60 months of follow-up, censoring before the cut-off could arise only through death, so the Kaplan–Meier estimate is close to the crude proportion. Both an unadjusted Cox model and a model adjusted for the 13 baseline covariates that varied within the phakic subgroup are reported. *p* values for both Cox models are two-sided Wald tests on the pooled log-hazard scale.

**Table 4:** Multivariable Predictors of Year-5 Visual Acuity and the Anatomical–Functional Relationship.

**A. Multivariable Model of Year-5 BCVA Change (R^2^ = 0.42)**				
**Predictor**	**β (Letters)**		**95% CI**	***p***
Baseline BCVA, per letter	+0.48		0.40 to 0.56	<0.001
Baseline EZ disruption	−4.2		−6.6 to −1.8	<0.001
PDR vs. moderate NPDR	−3.1		−5.4 to −0.8	0.008
Baseline CST, per 100 µm	−1.5		−2.1 to −0.9	<0.001
Age, per decade	−1.3		−2.4 to −0.2	0.02
HbA1c, per 1%	−0.9		−1.7 to −0.1	0.03
Treatment regimen (T&E vs. PRN)	−0.6		−2.5 to 1.3	0.53
Cumulative injections, per 5	+0.2		−0.5 to 0.9	0.58
**B. Segmented Regression of BCVA Change on CST Reduction**				
**Parameter**	**Estimate**		**95% CI**	***p***
Breakpoint, µm CST reduction	148		112 to 186	—
Slope below, letters/10 µm	0.61		0.41 to 0.81	<0.001
Slope above, letters/10 µm	0.04		−0.07 to 0.15	0.46
**C. Year-5 BCVA Change by Final CST Stratum**				
**Final CST stratum**	**T&E**	**PRN**	***p*** **(Within)**	***n***
<250 µm	+7.4	+7.9	0.68	268
250–299 µm	+7.0	+7.2	0.87	241
300–349 µm	+5.1	+5.4	0.85	163
≥350 µm	+1.9	+2.3	0.83	128
*p* for trend	<0.001			

Values in A are regression coefficients (letters). Regimen does not predict year-5 vision (A); a breakpoint occurs at 148 µm (B); final CST predicts vision but regimen does not differ within strata (C). BCVA = best-corrected visual acuity; CST = central subfield thickness; EZ = ellipsoid zone; NPDR = non-proliferative diabetic retinopathy; PDR = proliferative diabetic retinopathy.

## Data Availability

The de-identified analysis dataset and complete analysis code are deposited in Zenodo (https://doi.org/10.5281/zenodo.21297166). No direct or indirect identifiers are included; dates are expressed as intervals from baseline and the record does not permit re-identification.
